# REAL LIVES ACROSS THE GLOBE: INSIGHTS FROM THE GLOBAL PRIDE STUDY

**DOI:** 10.1093/geroni/igae098.1089

**Published:** 2024-12-31

**Authors:** Austin Oswald, Brian de Vries, Karen Fredriksen-Goldsen

**Affiliations:** Dalhousie University, Nova Scotia, Canada; San Francisco State University, San Francisco, California, United States; University of Washington, Seattle, Washington, United States

## Abstract

Research on the intersection of sexuality, gender, and aging is becoming increasingly global; however, scholars in the field rarely have the opportunity to collaborate across national boundaries. This hampers knowledge sharing and limits the global knowledge base on sexuality, gender, and aging. In this presentation, we will explore international and interdisciplinary perspectives on sexuality and gender across the lifespan. Individual and group interviews were conducted with collaborators of the Global Pride study, discussing the challenges and opportunities of global research at the intersection of sexuality, gender, and age. These interviews were video recorded and will be shared during this presentation. The video features global collaborators discussing the resources and cultural influences in various conceptual, qualitative, quantitative, and multi-method approaches for conducting sexuality, gender, and aging research. We will also address the unique cultural influences, dimensions, and dynamic nature of conducting cutting edge research with sexual and gender diverse (SGD) adults across the lifespan. Key insights include: 1) identifying similarities and differences across diverse cultural contexts, including variations in old age itself; 2) understanding the distinct realities and challenges faced by older adults in diverse SGD communities and the strengths and fortitude of these communities worldwide; 3) developing feasible strategies to advance SGD research, practice, and education within differing cultural, social, and political environments. By examining the risk and resilience of SGD older adults globally, we aim to illuminate how innovative research, practice and education can strengthen our understanding of aging in our increasingly diverse society.

